# Patient-reported outcome measures in oncology: a qualitative study of the healthcare professional’s perspective

**DOI:** 10.1007/s00520-021-06052-9

**Published:** 2021-03-02

**Authors:** Caitlin Graupner, S. O. Breukink, S. Mul, D. Claessens, A. H. M. Slok, M. L. Kimman

**Affiliations:** 1 Department of Surgery, Maastricht University Medical Centre, P. Debyelaan 25, 6229 HX Maastricht, The Netherlands; 2 School for Oncology and Developmental Biology (GROW), Maastricht University, Maastricht, The Netherlands; 3 School of Nutrition and Translational Research in Metabolism (NUTRIM), Maastricht University, Maastricht, The Netherlands; 4 Department of Family Medicine, Care and Public Health Research Institute (CAPHRI), Maastricht University, Maastricht, The Netherlands; 5 Department of Clinical Epidemiology and Medical Technology Assessment, Care and Public Health Research Institute (CAPHRI), Maastricht University Medical Centre, Maastricht, The Netherlands

**Keywords:** Patient-reported outcome measure, PROM, Caregiver perspectives, Oncology, Barriers and facilitators, Personalised care, Implementation science

## Abstract

**Background:**

In the last decades, the number of cancer survivors has increased significantly due to improved treatment and better detection of recurrence. This increased survival redirects the scope from survival towards optimising functional outcomes and improving health-related quality of life (HRQol). Functional and HRQoL outcomes can be assessed with patient-reported outcome measures (PROMs). However, the use of PROMs in daily oncological care is not common. This qualitative study investigates the barriers and facilitators of PROM use in an oncological setting, from the perspective of the healthcare professionals (HCPs).

**Methods:**

Individual semi-structured interviews were conducted among Dutch oncological HCPs. Barriers and facilitators of PROM implementation were identified on various levels of the healthcare system (i.e. level of the patient, individual professional, medical team, and healthcare organisation). Interviews were audio recorded and transcribed verbatim. Transcripts were manually analysed by two independent reviewers using a thematic approach. Identified barriers and facilitators were categorised into Grol and Wensing’s framework for changing healthcare practice.

**Results:**

Nineteen oncological HCPs working in academic and non-academic hospitals were interviewed. Barriers for PROM implementation were lack of good IT support, lack of knowledge on how to use PROMs, lack of time to complete and interpret PROMs, and a high administrative burden. PROM implementation can be facilitated by providing clear guidance regarding PROM interpretation, evidence that PROMs can save time, and stimulating multidisciplinary teamwork.

**Conclusion:**

From a HCP point of view, adequately functioning IT technology, sufficient knowledge on PROMs, and dedicated time during the consultation are essential for successful implementation of PROMs in oncological care. Additional local context-specific factors need to be thoroughly addressed.

## Introduction

Due to earlier detection and improved treatment, locoregional control and overall survival of cancer patients improved substantially in the last decades [1]. The increased survival enables the scope of daily oncological care to be redirected from survival-focused towards maintaining or increasing health-related quality of life (HRQoL) and functioning outcomes [2]. HRQoL and functioning can be reported by patients themselves using patient-reported outcome measures (PROMs). PROMs can be used to identify physical, psychological, and social needs, and monitor symptoms and treatment effects [3–5]. Routine use of PROMs can then facilitate personalised care and shared decision-making (SDM) [6, 7]*.*

Several oncological and non-oncological studies reported that the use of PROMs is associated with improved patient-physician communication, higher patient satisfaction, and improved symptom control. Results were most pronounced when feedback was provided to patient and healthcare professional (HCP) regarding the findings of the PROM [8–10].

Even though the benefits of PROMs have been shown, they are not yet an integral part of routine clinical care [11, 12]. Barriers to implementation can include individual, structural, and organisational factors. Reported reasons for clinicians not engaging in routine PROM use are a low familiarity with the concept of patient-reported outcomes, the lack of available validated questionnaires, and a potential loss of human touch between caregiver and patient [13, 14]. Furthermore, HCPs might be concerned that incorporating PROMs disturbs their workflow and augment their already existing administrative burden. Alternatively, a recent study in haematological care found that a strict commitment to the biomedical perspective rather than the patient’s own experience of the disease was also a factor that prevented the use of PROMs [15].

While many of these barriers are logically present in most settings, local context-specific factors need to be thoroughly examined before implementation. The context in which changes are needed to implement a novelty such as routine use of PROMs is complex and consists of various levels. In order to assess these complex contextual factors, Grol et al. developed a framework in which these levels are described [16]. All domains of the framework need to be considered when aiming for change: the economic and political context, the individual professional, the innovation itself, the organisational context, the social context, and the patient. Grol et al. described the importance of understanding these domains and urged to tailor implementation strategies to them [16, 17].

In the Netherlands, like many countries, PROMs are not yet part of routine clinical care. In line with Grol’s recommendations, we first need to better understand the perceived barriers and facilitators HCPs experience in the use of PROMs before implementation strategies can be developed. The aim of this study was therefore to identify barriers and facilitators for PROM implementation in the Dutch oncological setting through semi-structured interviews with HCPs.

## Methods

### Study design

A qualitative study was performed using a pragmatic approach [18]. A pragmatic approach, also referred to as descriptive, has a strong focus on practicality and aims to understand and describe a phenomenon, a process, or the perspectives of the people involved [19]. Specifically, we conducted semi-structured interviews with HCPs to gain a holistic understanding of barriers and facilitators for PROM implementation. The study is reported according to the Consolidated Criteria for Reporting Qualitative Research (COREQ) [20].

### Participants

HCPs working in oncological care in Dutch hospitals were invited to participate. A mixture of convenience and purposive sampling was used to recruit HCPs working in the field of colorectal, breast, and gynaecologic cancer, in academic and non-academic centres, small and large centres, and with at least some experience in using PROMs. We used the snowball method to identify other potential participants, by asking the HCPs if they knew any other relevant HCPs. The sample size was determined by the theoretical data saturation principle, i.e. interviews were stopped when no new information emerged from the interviews.

### Data collection

HCPs were interviewed alone or in a duo interview, based on availability of the HCPs. Interviews were performed face-to-face in the hospital where the HCPs worked or by phone. All interviews were conducted by one researcher (CG), who is a female medical doctor working as a clinical investigator, and trained in conducting interviews. The interviewer did not have a relationship with any of the HCPs and no other people were present during the interviews.

Interviews were guided by an interview topic guide (Appendix Table 3) that was based on the conceptual framework of Grol et al. concerning implementation of change in clinical practice [16]. The framework developed by Grol et al. provides an overview of six features that could influence change of medical practice and implementation of guidelines: (1) the organisational context, (2) the innovation itself, (3) the individual professional, (4) the patient, (5) the social context, and (6) the economic and political context (box 1). All interviews were audio recorded and transcribed verbatim. Transcripts were not returned to participants.

**Table:** Box 1. Conceptual framework by Grol et al. [16]

**- Organisational context**	
**○** This feature describes the organisation of care processes, staff, capacities, resources and structures when using a PROM in daily cancer care.	
**- Innovation itself**	
**○** This feature describes the opinion of the HCPs regarding the use of PROMs in daily cancer care. The focus will be on the advantages and disadvantages in practice, feasibility, credibility, accessibility and attractiveness of using PROMs.	
**- Individual professional**	
**○** This feature describes the perception of the HCPs regarding their own awareness, knowledge, attitude, motivation to change and behavioural routines regarding the use of PROMs in daily cancer care.	
**- Patient**	
**○** This feature describes the knowledge, the skills, the attitude and the compliance of patients regarding the completion of PROMs experienced by HCPs.	
**- Social context**	
**○** This feature describes the attitude of colleagues, the culture of the network, the collaboration (between colleagues and/or departments) and leadership in the organisation regarding the use of PROMs in daily cancer care experienced by HCPs.	
**- Economic and political context**	
**○** This feature describes the financial arrangements, regulations and policies regarding the implementation of PROMs in daily cancer care.	

### Data analysis

Two researchers (CG and SM) analysed the data manually using a thematic approach [21]. First, the researchers independently read the first five transcripts and coded them line-by-line. A deductive coding strategy was used, assigning predefined codes to various words, phrases, or paragraphs. The codes included the subthemes of the Grol framework, but new codes could also be introduced. Second, the researchers compared assigned codes and resolved coding discrepancies by discussing each conflicting code. Any new codes identified were discussed and, if appropriate, added to the coding scheme. CG and SM then analysed all codes and associated fragments and grouped similar concepts together into the main themes within Grol’s framework, and assigned them to be a barrier and/or facilitator. If appropriate, new themes were added. Data were managed using Microsoft Excel© software.

## Results

### Study participants and context

Nineteen HCPs from academic and non-academic centres participated in 18 interviews (see Table 1). HCPs included colorectal surgeons, breast surgeons, gynaecologists, specialised oncology nurses, and an oncologist. In addition, a case manager and an IT specialist were identified by participants because of their expertise in oncological PROM implementation in the hospital.

**Table 1:** Characteristics of participants

Characteristic	*N* (%)
Specialty	
Breast cancer	5 (26)
Gynaecological cancer	4 (21)
Colorectal cancer	7 (37)
Other	3 (16)
Type of healthcare professional	
Medical specialist	7 (37)
(specialised) nurse	10 (53)
Other	2 (11)
Centre	
Academic	8 (42)
Non-academic	11 (58)
Type of interview	
Face-to-face	8 (44)
Telephone	10 (56)
Average duration in minutes (range)	22 (9–42)

The use of PROMs, more specifically the collection method, timing of data collection, and data analysis, varied across hospitals and settings. Some hospitals made use of online surveys to collect PROM data (e.g. a link sent via email, a unique code to access a separate online system, or a handheld device in the waiting room), and others used ‘paper-and-pen’ questionnaires sent to the patient’s home. The timing of patient-reported outcome (PRO) collection in relation to hospital visits also differed between the hospitals and specialties, and ranged from a few days before the outpatient clinic visit to a few weeks beforehand. For example, in one hospital, a tablet with several PROMs is given to patients in the waiting room, before their consultation. Furthermore, some hospitals collect PROMs every 3 months during treatment and follow-up, while in others, patients are asked to complete PROMs at 6-month intervals for the first 2 years and annually up to 5 years post treatment.

Mainly specialised oncological nurses were involved in the PROM collection and used the results during consultations, whereas medical specialists hardly used PROMs at all. Several PROMs were used, with the most commonly used PROMs being the European Organisation for Research and Treatment of Cancer (EORTC QLQ-C30), the distress thermometer, and the low anterior resection syndrome score (LARS score) [22–24]. Four participants did not have experiences in using PROMs in daily care, but their departments were in the process of implementation of PROMs at the time of the interviews.

### Barriers and facilitators

According to Grol’s framework, we aimed to categorise the identified barriers and facilitators into five themes: (1) innovation, (2) healthcare professionals, (3) patients, (4) social context, and (5) economic context and regulations. No barriers and facilitators were found in the theme of economic context and regulations. HCPs were generally not closely involved in the hospital-specific financial arrangements and regulations regarding PROMs and felt therefore unequipped to provide information about this. No additional themes were identified to be added to the framework of Grol et al. All of the themes contain factors that can act as a barrier, a facilitator, or both at the same time. For example, a PROM can save time during the consultation because the patient’s problems are quickly identified, yet when many problems are detected, discussing all can cause an extension in consultation time. Results and illustrative quotes are summarised in Table 2.

**Table 2:** Summary of findings presented in Grol’s framework

Theme/subtheme	Predominantly barriers or facilitators	Results	
Innovation			
Advantages in practice	Facilitators	Problems easily detected, saving time, targeted care, focus on well-being, alignment perspectives doctor and patient, better feedback	“When you have that list, you can easily recognize any problems and I see them beforehand, so I can act on it immediately” (nurse)
Feasibility	Barriers	Time constraints, list not visible, no graphic results, high workload	“Next to all the extra things we have to do and discuss during consultation, it’s just almost impossible to attain. It’s too much” (doctor)
Credibility	Barriers	No scientific proof of value	“They [doctors] do not want to use it, since there is not enough evidence regarding the added value in oncology treatment” (doctor)
Accessibility	Barriers	Different medical specialties are not able see each other’s outcome data, too long, low literacy	“You always have to open a second program to see if the patient needs to fill out a PROM for this consultation. It doesn’t work” (nurse)
Attractiveness	Barriers	No quick overview	“It has to be insightful fairly quickly. What is the patient’s progress is it getting better or worse? But that is missing now” (nurse)
Individual professional			
Awareness	Mixed	Knowledge of existence	“Ehm, well we know they are out there” (nurse)
Knowledge	Barriers	Lack of knowledge on which PROMs are used, lack of interpretation	“We did not know what questionnaires patients filled out. Not until you [the researcher] send us examples of the questionnaires. It is insightful to know what we are actually asking our patients” (nurse)
Attitude	Facilitators	Importance of monitoring quality of life, PROMS are valuable, addition to healthcare	“But we also have to include quality of life. That is the most important aspect” (nurse)
	Barriers	Administered too frequently, no functional computer system, difficult to implement	“I feel like we burden patients with it” (nurse)
Motivation to change	Facilitators	Express motivation, express benefits	“And we ourselves, of course, absolutely had the motivation to start” (nurse)
	Barriers	Too little time, no difference in treatment	“Because people are unfamiliar with it. And unknown is unloved” (nurse) “I can pretend that it’s all good, but I think that.. I hope that we will get rid of this [PROMs] in a couple of years” (nurse)
Behavioural routines	Facilitators	Years of experience in the team	“We have been working with PROMs for quite a long time actually” (nurse)
	Barriers	Not used and entirely forgotten	“But we have to say, my colleague and I found out that after a while the use of PROMs diminished. That you quickly forget” (nurse)
Patient			
Knowledge	Barriers	Patients do not see benefits	“Ehm well, they don’t see the benefit of it. You wouldn’t fill in a dozen of lists if you never heard anything back” (doctor)
Skills	Mixed	No internet/computer skills Low vs. high literacy	“What you’re dealing with is dependent on the patient’s level. One patient can oversee a page and answer the questions easily. Another already has difficulty with one question on a page” (doctor)
Attitude	Barriers	Too much of a burden, repeatedly asked by different clinicians, results not reported back	“Because they do fill them in sometimes, but nothing is done with it. So why should they?” (nurse)
Compliance	Mixed	Initial compliance good, diminished compliance over time	“Well the first PROM is completed, but completion rates drop over time” (nurse)
Social context			
Opinion of colleagues	Facilitators	Willingness to improve care, value and benefits recognised	“And that the willingness is great, to ehm keep improving care” (nurse)
	Barriers	Unfamiliar with PROMs, aversion because lack of proof of value, unwillingness to do it themselves	“Because I feel that a lot of people think it is difficult. You know, they don’t quite know what it is, it’s new so it is often a bit scary as well” (doctor)
Culture of the network	Facilitators	Teamwork, motivated towards improvement	“Doctors certainly not. It’s mostly nurses working in surgery and specialized breast care nurses“ (nurse)
	Barriers	Skepticism about utility or colleagues	“Everybody is, of course, afraid of more administrative burden coming towards you. That is a thing” (doctor)
Collaboration	Facilitators	Teamwork, division of tasks, mutual transparency	“So we are very aware of it, and deliberately working on it as a team, also multidisciplinary” (nurse)
	Barriers	No teamwork, no collaboration between different locations	“And we consult with colleagues from other locations, and they say: well, we are not doing anything with that actually. So that ehm, does not motivate us” (nurse)
Leadership	Facilitators	Initiative, guiding implementation	“From the specialists, a departmental manager that supports it” (doctor)

### Innovation

Both doctors and nurses reported that PROMs helped them in the detection of health problems and provided guidance during the consultation. When PROMs were collected regularly, they also provided information about the dynamics of a patient’s quality of life, and changes in health could be detected more easily (e.g. sudden increase in fatigue). This generally saved them time during the consultation, which was seen as an advantage of PROMs.

Yet, use of a PROM in clinical practice was also described as time consuming. Results of completed PROMs have to be consulted by HCPs in order to discuss them during the patient’s clinic visit. Some worried that not enough time was available during the consultation to discuss all the problems that were identified by the PROM. The current workload and administrative burden (without the use of PROMs) of HCPs was already experienced as high. HCPs did express that a solid electronic system that displays the data in a comprehensible way would make the use of PROMs more feasible. Furthermore, if the time interval between the completion of a PROM and an actual visit at the outpatient clinic was too long (e.g. more than 4 weeks), the outcome of the PROM might be less relevant to the current situation of the patient. This was considered an important barrier.

Opinions differed on the ideal length of a PROM. A comprehensive questionnaire can provide a lot of information but will decrease the usability, whereas a short questionnaire perhaps provides (too) little information for the amount of effort needed from patients to complete the questionnaire. Hence, the length of a PROM is likely to influence the implementability.

### Healthcare professionals

Several HCPs stated that more knowledge about the content of the PROMs and how to interpret them would benefit the uptake of PROMs.

Not all HCPs had knowledge on how to score and interpret the results. It is often not clear whether a specific score is ‘good’ or ‘bad’, what a change in score means, and when it is clinically relevant. It was stated that there was little to no education about the interpretation of the results when the PROMs were being implemented in their care pathway. Easy-to-access training is generally lacking and therefore HCPs need to acquire this knowledge themselves. Hence, while most participants acknowledged that monitoring outcomes such as functioning and HRQoL could have benefits, correct monitoring of these outcomes was found to be difficult.

An important drive to change the ways PROMs are being used is an IT system that better supports HCPs during consultations. For example, HCPs expressed the need for easy access to the PROM data and display of the results in a way that is easy to understand for both patient and professional.

In some hospitals, PROMs were only used for benchmarking. Results were not returned to the HCP or patient and could therefore not be used in daily clinical practice. Finally, a few HCPs did not see any reason to use PROMs in their consultations since they believe either that patients would come to them if they experienced any problems, or they already discuss all PROM content. Some of the HCPs are convinced that they already cover all subjects that are mentioned in the PROMs in their regular consultation.

### Patient-related factors

HCPs emphasised the importance of clear information for the patient. HCPs have received feedback from patients that they do not understand why PROMs are used and that this lowers their motivation to complete a PROM. They experienced that patients were much more willing to fill out a PROM when they were properly informed about its purpose. Furthermore, the increased attention for the patient’s wellbeing was much appreciated. Besides, the person who invites the patient to complete a PROM seems to influence completion rates. Nurses felt that patients were more willing to complete a PROM when it was the doctor who invited them. Feedback during consultation about the results of the questionnaire was also essential for patients according to the HCPs.

In some hospitals, there is a lack of coordination and communication between departments regarding PROM collection. As a result, a patient may be asked to complete (the same or similar) PROMs for different oncological departments within a short timeframe. For example, in one hospital, the same PROM is sent to patients in both the surgery and radiotherapy department only 2 weeks apart from each other, which can be confusing to the patient and may lead to the second PROM not being returned.

Other barriers reported by patients to HCPs were that the questionnaires were too long, taking too much time to complete, that there were no possibilities for personal input, and that the questionnaires had to be completed electronically. Especially patients who experience difficulty working on the internet, or do not have easy access to a computer or tablet, experience barriers with using PROMs in this way. Furthermore, according to HCPs, patients felt it was a burden to complete a questionnaire when they actually did not experience any complaints and were feeling well.

### Social context

In general, the interviews revealed that the added value of PROMs was recognised by HCPs and that there was an overall willingness to improve the current healthcare system. It was expressed that HCPs should work together as a team with multiple disciplines and prioritise the patient’s needs in PROM implementation.

However, HCPs felt that other colleagues were still sceptical about the use of PROMs. They feel that there is not yet enough evidence for the added value of PROMs. Some of the nurses reported that doctors generally do want to use PROMs in the clinic, but do not want to use them during their own consultations. According to these nurses, doctors prefer that PROMs are used during the nurses’ consultations. Some HCPs expressed concerns about the ability of colleagues to correctly use (and interpret) PROMs, due to limited knowledge and education about PROMs. Another frequently mentioned barrier to the use of PROMs is the inability to share PROM results between departments. A shared system can ensure a patient does not need to complete the same PROM for different specialities.

## Discussion

In the Netherlands, PROM implementation is not centrally regulated and HCPs and hospitals can apply their own approach. This was reflected in this qualitative study conducted in the Dutch oncological care setting aimed to identify barriers and facilitators of PROM implementation. Experiences of the participants varied greatly, but did not seem to differ between academic and non-academic centres. Rather, experience with the use of PROMs and stage of implementation in the healthcare centre influenced experiences. In most participating centres, PROM use in routine practice was being pilot tested but not yet widely and fully implemented. The interview with an IT specialist provided a specialist view on IT solutions. The wide range of experiences of participants helped identify various barriers and facilitators. The most commonly mentioned factors that influenced the use of PROMs in daily cancer care were use of IT technology, knowledge of PROMs by both patient and HCP, and time. HCPs brought up these factors both in terms of facilitators and barriers. For example, an adequate IT solution that assists in retrieving, summarizing, and interpreting PROM data could result in saving time before and during the consultation for the HCP and could potentially lead to better patient care. At the same time, if there is no such IT solution, it would be considered a barrier to PROM implementation and PROM use could negatively influence patient care. All experienced barriers and facilitators were interrelated and occurred at multiple levels of the healthcare system.

Our findings are congruent with other studies evaluating barriers and facilitators of implementation of PROMS in other settings [15, 25]. The study of Hansen et al. reported on the lack of time experienced by nurses. Due to the high workload, nurses had to prioritise patient flow at the outpatient clinic, meaning that mandatory tasks during patient care were prioritised over discussing PROM results so patients were not unduly delayed by waiting times. Another overlapping theme addressed by Hansen et al. was the lack of education. There was a strong desire among the nurses for a standardized protocol on how to use the PROMs [15]. Turner et al. found that being unconvinced of the benefits of PROMs, lack of integration into electronic patient charts or not easy in use, and lack of time were barriers experienced by general practitioners in the implementation of PROMs [25]. Hence, the literature is agreeing on potential barriers and facilitators for PROM implementation, providing us with in-depth knowledge on possible ways forward.

Potential limitations of our study need to be addressed. First, member checks were not performed due to the time investment this would have required from the participants. Hence, participants could not agree or disagree with the researchers’ interpretation of their experiences, and classification into barriers and facilitators, nor the (sub)themes. In qualitative research, member checking is one way to ensure credibility and enhance the trustworthiness of the study [26]. Second, undoubtedly, the researcher brings her own theoretical and methodological expertise to the interview and data interpretation. However, by using Grol’s detailed framework, analyzing each participants interview separately (rather than synthesizing the results of the interviews), and presenting findings on a descriptive level using the participants’ words as much as possible, the potential impact and subjective interpretation of the researcher are expected to be limited. In addition, methodological rigor was maintained by performing the analysis and categorisation by two independent researchers, who subsequently compared and discussed their findings, and a review of the categorisation by a third independent experienced researcher. Yet, a residual element of subjectivity cannot be ruled out in any qualitative analysis.

Finally, this study focussed on the perspective of HCPs on PROMs. Therefore, the barriers and facilitators at the patient level should be interpreted with caution.

A strength of this study is the use of the framework by Grol et al. as a basis for our evaluation [16]. Using the framework helps us to gain a thorough understanding of on-going issues and benefits of PROM use in daily oncological practice. Furthermore, HCPs worked both in academic and non-academic hospitals, in different oncological departments, and with different levels of experience with PROMs. In this way, we gained a broad overview and therefore the results are likely to be generalizable to a broader Dutch oncological setting. Although there was a lot of variation in settings, type of HCPs, and PROM use, we are confident that our sampling approach identified the most important barriers and facilitators and that data saturation was reached. No new information occurred in the last five interviews. Nevertheless, adequate PROM implementation depends highly on the local context and thus the findings from our study should always be placed in that perspective.

Further research on PROM implementation should focus on the perspective of patients. They need to be engaged in implementation interventions, hence insight into their views on the advantages and disadvantages of PROMs, and facilitators for PROM use, is essential. Furthermore, IT systems should be studied and optimised as an important barrier/facilitator to implementation of PROMS. Presenting results in a visual format embedded in a patient’s electronic system and easy-to-access training for HCPs and patients on using PROMs are expected to promote meaningful use. Research is also needed on sustaining the use of PROMs, for example, what changes in the organisational culture could facilitate PROMs becoming part of routine practice. Moreover, standardizing outcomes and instruments used, following Core Outcome Sets (COS) such as developed by the International Consortium for Health Outcome Measurement (ICHOM), can potentially reduce the burden to clinicians and patients. The recommended PROMs in a COS are validated and usually well-known [27]. Finally, aligning multiple purposes of PROMs is also a major challenge. While PROMs are regularly designed to improve care in clinical practice, they can also be used as performance measures. Further work is needed to investigate the potential role of PROMs as performance measures, as they speak to other important stakeholders to implementation.

## Conclusion

This qualitative study provided a broad overview of the current problems of PROMs in oncological care in the Netherlands. There is no simple solution to address the difficulty of PROM implementation. Knowledge regarding the local barriers and facilitators is essential when further implementing PROMs. An IT solution that displays and shares data in a comprehensible way for patients and HCPs, visible for all departments within the hospital, could facilitate PROM implementation. In addition, it is important to provide evidence on the added value of PROMs and training in the use and interpretation of PROMs. To successfully implement PROMs in daily oncological care, these barriers and facilitators, as well as additional local context-specific factors, need to be thoroughly addressed.

## Appendix

**Table 3:** Interview topic guide based on the framework of Grol et al. [16]

Interview guide	
**Characteristics of interview** Semi-structured individual interview Approximately 30-60 minutes Participant informed about purpose of interview Audio recorded	
**Purpose of interview** To obtain an overview of the barriers and facilitators experienced by healthcare professionals in the use of PROMs in daily colorectal, breast and gynaecological oncological care.	
**Topics, questions and potential sub-questions** Can you introduce yourself briefly? Tell us your name, your profession and your workplace? Are PROMs being used in your clinic? If so: tell us something about the use of PROMs in your clinic: - How are PROMs collected? - Which PROMs are asked to complete? - How many times are patients being asked to complete a questionnaire and how often does this actual happen? - Is feedback of the completed questionnaires being provided to yourself and the patients? - How is the organisation of PROMs financed? Do you have experience in using PROMs yourself? What is your opinion regarding PROMs? And how is that of your colleagues? Are you satisfied with the way the PROMs are organised in your clinic? - What are ways to improve this organisation? How does using PROMs influence your consultation? What do the patients tell you about the use of PROMs? - Are patient willing to participate? Are there any other issues you want to say about the subject that we have not discussed yet?	

## Abbreviations

PRO: Patient-reported outcome
PROM: Patient-reported outcome measure
HRQoL: Health-related quality of life
